# Thrombospondin-1 Interacts with *Trypanosoma cruzi* Surface Calreticulin to Enhance Cellular Infection

**DOI:** 10.1371/journal.pone.0040614

**Published:** 2012-07-11

**Authors:** Candice A. Johnson, Yulia Y. Kleshchenko, Adaeze O. Ikejiani, Aniekanabasi N. Udoko, Tatiana C. Cardenas, Siddharth Pratap, Mark A. Duquette, Maria F. Lima, Jack Lawler, Fernando Villalta, Pius N. Nde

**Affiliations:** 1 Department of Microbiology and Immunology, Meharry Medical College, Nashville, Tennessee, United States of America; 2 U.S. Military Malaria Vaccine Program, Naval Medical Research Center, Silver Spring, Maryland, United States of America; 3 Department of Pathology, Beth Israel Deaconess Medical Center, Boston, Massachusetts, United States of America; INSERM U1094, University of Limoges School of Medicine, France

## Abstract

*Trypanosoma cruzi* causes Chagas disease, which is a neglected tropical disease that produces severe pathology and mortality. The mechanisms by which the parasite invades cells are not well elucidated. We recently reported that *T. cruzi* up-regulates the expression of thrombospondin-1 (TSP-1) to enhance the process of cellular invasion. Here we characterize a novel TSP-1 interaction with *T. cruzi* that enhances cellular infection. We show that labeled TSP-1 interacts specifically with the surface of *T. cruzi* trypomastigotes. We used TSP-1 to pull down interacting parasite surface proteins that were identified by mass spectrometry. We also show that full length TSP-1 and the N-terminal domain of TSP-1 (NTSP) interact with *T. cruzi* surface calreticulin (TcCRT) and other surface proteins. Pre-exposure of recombinant NTSP or TSP-1 to *T. cruzi* significantly enhances cellular infection of wild type mouse embryo fibroblasts (MEF) compared to the C-terminal domain of TSP-1, E3T3C1. In addition, blocking TcCRT with antibodies significantly inhibits the enhancement of cellular infection mediated by the TcCRT-TSP-1 interaction. Taken together, our findings indicate that TSP-1 interacts with TcCRT on the surface of *T. cruzi* through the NTSP domain and that this interaction enhances cellular infection. Thus surface TcCRT is a virulent factor that enhances the pathogenesis of *T. cruzi* infection through TSP-1, which is up-regulated by the parasite.

## **Introduction**

*Trypanosoma cruzi* is an obligate intracellular protozoan parasite, which causes the debilitating Chagas heart disease. Chagas disease, which was once thought to be an exotic disease confined to endemic regions of Latin America, has now gone global becoming a new worldwide challenge [1], [2]. We have reported that the parasite up-regulates the expression of TSP-1 and other extracellular matrix (ECM) proteins in human coronary artery smooth muscle cells in order to enhance the process of cellular invasion [3], [4]. It has been suggested that *T. cruzi* trypomastigotes bind to several extracellular matrix components such as laminin [5], fibronectin [6], collagen [7] and human galectin-3 [8], [9] to increase cellular infection through not well understood mechanisms.

Thrombospondins have been described as “matricellular proteins” because they play a role in regulating cellular responses and ECM remodeling in the pericellular microenvironment but they are non-essential components of the mature matrix fibrils [10], [11]. The role of TSP-1 *in vitro* and *in vivo* is complex and context specific, because it interacts with a wide array of cellular proteins. TSP-1 is a large homotrimeric glycoprotein containing several domains that can bind to cell surface receptors and extracellular molecules [12]. TSP-1 is composed of several characterized distinct domains including the N-terminal heparin binding domain (NTSP), procollagen region, type 1, 2 and 3 repeats and a C-terminal domain [13]. The molecule also contains highly conserved Epidermal Growth Factor (EGF) repeats, type 3 repeats and a C-terminal domain, which includes the signature domain [14] that can interact with integrins and CD47 [15], [16]. The C-terminal domain of the thrombospondin family is highly conserved compared to the N-terminal domain, which is different for each thrombospondin isoform. Calreticulin (CRT) is a major intracellular well conserved calcium-binding chaperone, which was identified in skeletal muscle [17] and is present in the cells of all higher organisms except erythrocytes [18]–[20]. Numerous reports have implicated CRT in several cellular functions and the molecule has significant non-endoplasmic reticulum functions in normal physiology and human disease status [21]. CRT has also been described in some parasite species such as *Shistosoma mansoni*, *Onchocerca volvulus, Necator americanus, Leishmania donovani and Plasmodium falciparum.* However, the role that this protein might play in the parasite’s interaction with the host immediate microenvironment remains unknown [22]–[24]. In *T. cruzi*, it has been suggested that CRT expressed on the parasite [23], [25] could play a role in enabling the pathogen to evade the host immune response by interacting with the C1q component of complement [23].

The mechanism by which the parasite CRT interacts with host proteins to enhance the process of cellular invasion remains unknown. In this study, we hypothesize that *T. cruzi* uses its surface TcCRT to exploit matricellular proteins regulated by the parasite to enhance cellular infection. Pre-incubation of *T. cruzi* trypomastigotes with TSP-1 or NTSP significantly enhances the infection of wild type MEF compared to TSP-1 KO MEF. Blocking the TcCRT-TSP-1 interaction by pre-incubating the parasites with TcCRT antibodies significantly inhibits the enhancement of cellular infection mediated by the TcCRT-TSP-1 interaction. Here we show that host TSP-1 interacts with TcCRT expressed on the surface of the parasite to enhance cellular infection.

## Results

### Labeled TSP-1 Binds Specifically to the Surface of Invasive *T. cruzi* Trypomastigotes

**Figure 1:**
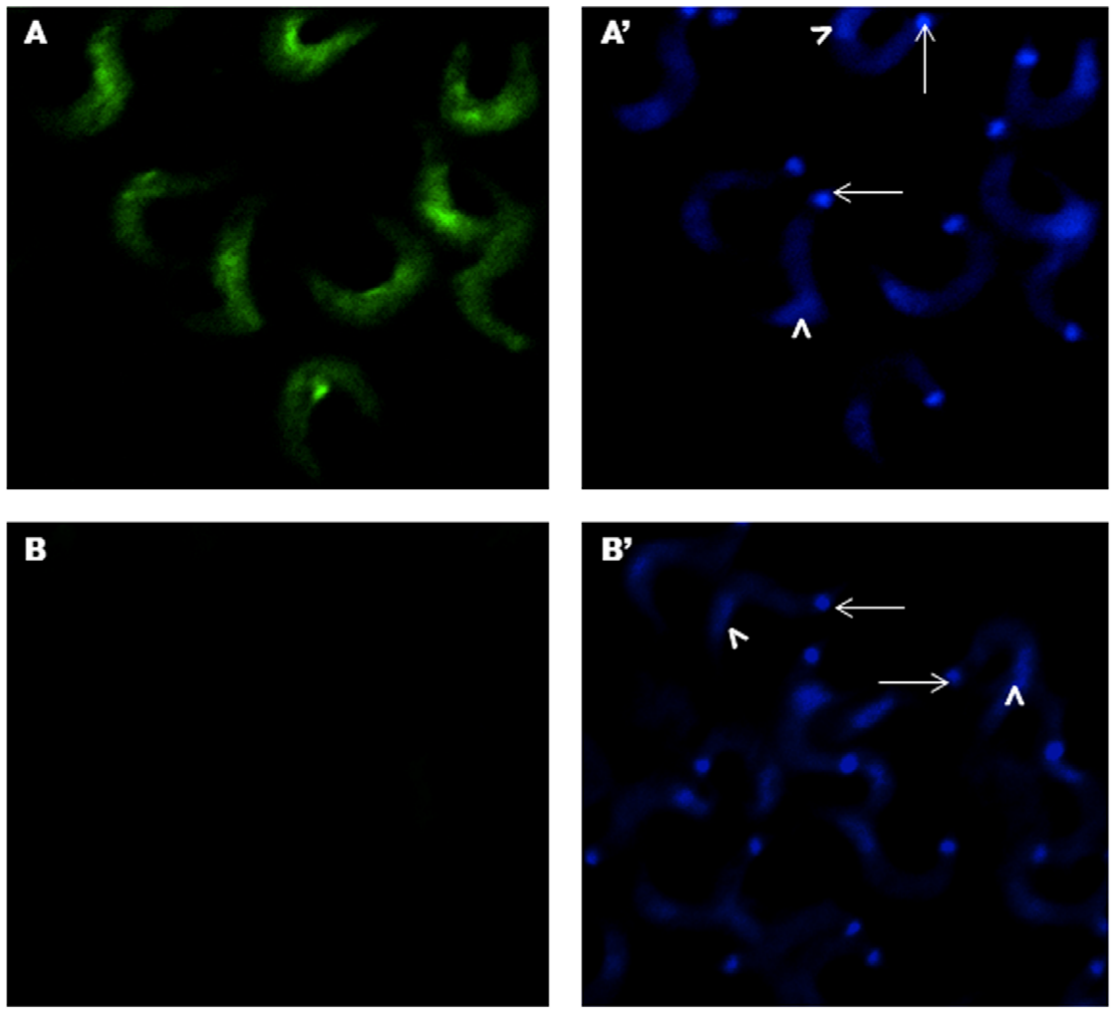
TSP-1 binds to the surface of infectious *T. cruzi* trypomastigotes. A , Alexa-488 labeled TSP-1 binds to the surface of paraformaldehyde fixed *T. cruzi* trypomastigotes as seen by fluorescence microscopy. **A’**, DAPI stained preparation of A showing parasite nuclear DNA (arrow heads) and k-DNA (arrows). **B**, Binding of Alexa-488 labeled TSP-1 to paraformaldehyde fixed *T. cruzi* trypomastigotes is inhibited by 100X unlabeled TSP-1. **B’**, DAPI stained preparation of B showing parasite nuclear DNA (arrow heads) and k-DNA (arrows). This figure shows the results of a representative experiment selected from three independent experiments with similar results.

We have previously shown that *T. cruzi* increases both the transcript and protein levels of human TSP-1 in cells to enhance the process of cellular infection. The increase in cellular infection was reversed by down-regulation of TSP-1 expression by RNAi [3]. In order to investigate if TSP-1 up-regulated by *T. cruzi* binds to the parasite’s surface to facilitate the process of cellular infection, we exposed paraformaldehyde fixed invasive trypomastigotes to recombinant TSP-1 labeled with Alexa Fluor 488. We observed that labeled TSP-1 binds to the surface of invasive *T. cruzi* trypomastigotes (Figure 1A); the parasite nuclear and kinetoplast DNA were visualized with DAPI staining (Figure 1A’). The binding of labeled TSP-1 was localized to the surface of the middle body of the trypomastigotes and was reduced towards the edges of the parasite (Figure 1A). Binding was abolished when the parasites were pre-incubated with unlabeled 100-fold excess of recombinant TSP-1 before exposure to Alexa-488 labeled TSP-1 (Figure 1B). The lack of fluorescence was not due to the absence of parasites since parasite nuclear and kinetoplast DNA stained with DAPI were microscopically observed (Figure 1B’), indicating that the binding of labeled TSP-1 to the surface of *T. cruzi* trypomastigotes is specific.

### Co-precipitation, Cloning and Purification of *T. cruzi* –TSP-1 Interacting Protein

**Figure 2:**
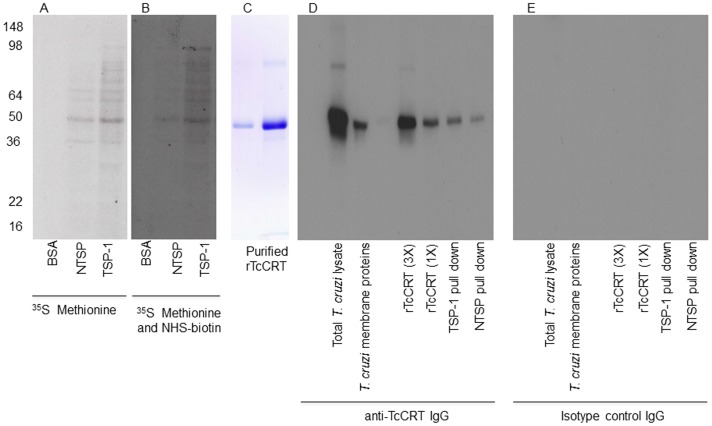
TSP-1 or NTSP pulls down surface TcCRT. **2A**, TSP-1 or NTSP pulls down a metabolically labeled *T. cruzi* 48 kDa protein whereas BSA control does not. Extracted ^35^S-methionine labeled trypomastigote membrane fraction was incubated with NTSP, TSP-1 or BSA coated Dynabeads to pull down interacting proteins. The Pull downs were separated by SDS polyacrylamide gels, transferred onto nitrocellulose (NC) membranes and developed by autoradiography. **2B**, NTSP or TSP-1 pulls down double labeled *T. cruzi* 48 kDa surface protein whereas BSA control does not. Extracted ^35^S-methionine and NHS-biotin double labeled surface proteins of *T. cruzi* interacting with NTSP, TSP-1 or BSA coated Dynabeads were recruited with streptavidin Dynabeads, separated by SDS polyacrylamide gel, transferred onto NC membranes and developed by autoradiography. **2C**, Purification of rTcCRT. Coommassie blue stain of two eluted fractions of affinity purified His-tagged rTcCRT. **2D**, TSP-1 or NTSP pulls down a TcCRT as evidenced by immunoblotting using anti-TcCRT IgG. **2E**, Blot of figure 2D was stripped and reprobed with an isotype control antibody. This figure shows the results from a representative experiment of three independent experiments performed with similar results.

The specific binding of labeled TSP-1 to the surface of *T. cruzi* trypomastigotes indicated that there are molecules on the surface of the parasite that interact specifically with TSP-1. In order to identify the parasite surface molecules that interact with TSP-1, we performed co-precipitation assays. TSP-1, NTSP or BSA were coupled to tosylactivated dynabeads M-280 followed by incubation with a solubilized membrane fraction of ^35^S-methione metabolically labeled infectious *T. cruzi* trypomastigotes. Metabolically labeled parasite proteins interacting with TSP-1, NTSP or BSA as control protein were separated on a 12.5% acrylamide gel, transferred to nitrocellulose membranes and exposed to X-ray film. We observed that NTSP pulled down a protein band of 48 KDa and minor protein bands of 38, 60, and 68 KDa (Figure 2A). Full length TSP-1 also pulled down the same 48 KDa protein band as NTSP as well as other minor proteins between 60 to 98 KDa including 30 and 38 kDa proteins (Figure 2A). To determine whether the TSP-1 or NTSP co-precipitated trypanosome interacting proteins were parasite surface proteins, we first metabolically labeled trypomastigotes with ^35^S-methionine followed by a second labeling with NHS-biotin, which does not penetrate the organism. Then, the double labeled and solubilized *T. cruzi* trypomastigote membrane proteins were co-precipitated with TSP-1, NTSP or BSA. The ^35^S methionine and NHS-biotin double labeled membrane parasite proteins interacting with TSP-1, NTSP or BSA were pulled down with dynabeads M-280 streptavidin, separated on SDS-PAGE, transferred onto nitrocellulose membranes and developed by autoradiography (Figure 2B). The autoradiogram showed that a 48 kDa parasite membrane protein was pulled down by both TSP-1 and NTSP. This indicates that both NTSP and TSP-1 interact with a 48 kDa *T. cruzi* trypomastigote membrane protein. Furthermore, the fact that these proteins were labeled by NHS-biotin indicates that the interacting membrane proteins are located on the surface of the parasite. This co-precipitation experiment was repeated using solubilized unlabeled *T. cruzi* membrane proteins and the eluted proteins were separated and analyzed by mass spectrometry. LC-MS/MS analysis indicated that the NTSP co-precipitated proteins include heat shock proteins (HSPs) 40, 60, 70, 85 kDa and other non-HSP including TcCRT (Nde *et al.,* unpublished). TcCRT had 16% protein coverage with 11 peptide spectral counts indicating a very high degree of confidence in its identification by LC-MS/MS. Therefore, we decided to focus on TcCRT in this study, since it is a parasite surface protein that interacts with both NTSP and TSP-1, which plays an important role in host ECM and cellular microenvironment.

In order to clone and purify rTcCRT to generate antibodies to characterize the TcCRT-TSP1 interaction, total *T. cruzi* RNA purified from invasive trypomastigotes was reverse transcribed into cDNA and the TcCRT cDNA was amplified, subcloned into pGS21a, which is an expression vector equipped with an N-terminal His-tag. The overexpressed recombinant protein was affinity purified as an N-terminal histidine tagged protein (Figure 2C). The antibodies generated using the rTcCRT reacted strongly with native TcCRT in the total parasite lysate, *T. cruzi* membrane protein preparation, rTcCRT, and TcCRT pulled down by both TSP-1 and NTSP (Figure 2D) compared to the isotype control (Figure 2E). TcCRT IgG also shows a weak reaction with a possible protein of approximately 85 kDa.

**Figure 3:**
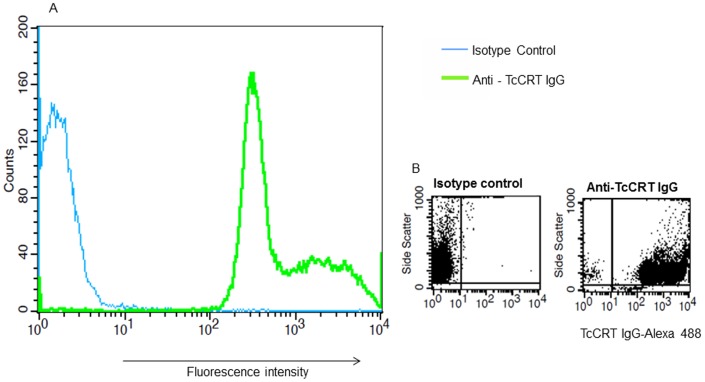
TcCRT is expressed on the surface of *T. cruzi* trypomastigotes. A , Alexa labeled TcCRT IgG binds to the surface of *T. cruzi* trypomastigotes as revealed by flow cytometry. Shift in the peak of fluorescence indicates that parasite surface is stained with labeled TcCRT IgG. **B**, Fluorescence intensity of parasites stained with labeled isotype control (left panel) and anti-TcCRT IgG (right panel) showing the high percentage (99.2%) of stained parasites. This figure shows the results of a representative experiment selected from three independent experiments with similar results.

To detect the expression of TcCRT on the surface of the parasite, we incubated *T. cruzi* trypomastigotes with Alexa Fluor 488-labeled anti-TcCRT IgG or isotype labeled control and analyzed the samples using flow cytometry (Figure 3). We observed that 99.2% of the parasites were stained by the labeled anti-TcCRT IgG compared to the labeled isotype control. Figure 3A shows a shift in the peak of fluorescence intensity indicating that the parasite surface was stained with the labeled antibody. Figure 3B shows two parameter graphs of parasites stained with labeled isotype control (left panel) and labeled anti-TcCRT (right panel) respectively.

### Role of TcCRT and TSP1 in the Cellular Infection Process by *T. cruzi*

**Figure 4:**
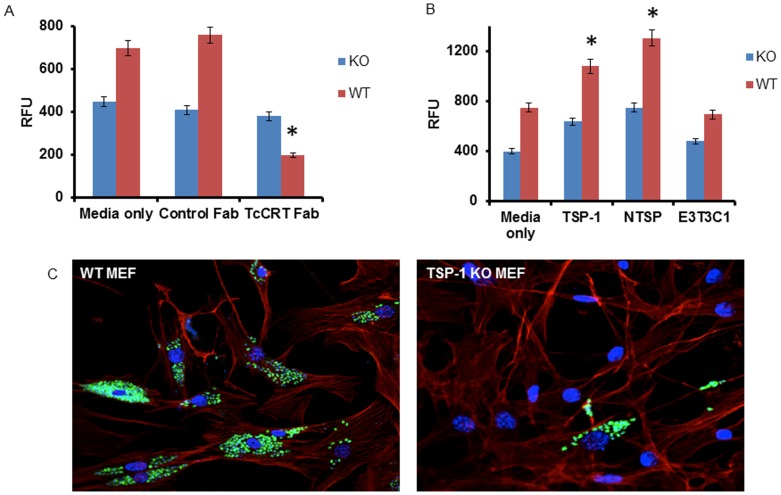
Roles of TcCRT and TSP-1 in cellular infection. **A**, TcCRT Fab blocks trypanosome cellular infection**.** Transgenic *T. cruzi* trypomastigotes expressing GFP were pretreated with either monovalent Fab fractions of anti-TcCRT or with monovalent Fab fractions of an isotype control and exposed to either WT or TSP-1 KO MEF for 72 hours. The infection was determined fluorimetrically. **B**, TSP-1 and NTSP enhance trypanosome infection but not E3T3C1. Pre-incubation of transgenic *T. cruzi* trypomastigotes expressing GFP with endotoxin free TSP-1 or NTSP enhances cellular infection of WT MEF compared to TSP-1 KO MEF. Pre-incubation of transgenic *T. cruzi* trypomastigotes expressing GFP with endotoxin free E3C3T1 does not significantly affect cellular infection of WT or TSP-1 KO MEF. Bars represent the means of results from triplicate samples of one representative experiment (±1 S.D.) selected from three independent experiments with similar results. *Significant differences between WT and KO MEF (P<0.05). **C,** The absence of TSP-1 expression reduces cellular infection. GFP expressing transgenic trypomastigotes exposed to TSP-1 KO MEF (Fig. 4C right panel) for 72 hours sustained less infection than WT MEF (Fig. 4C left panel), as observed by fluorescence microscopy. Cell monolayers were stained with DAPI (blue nuclei), Alexa fluor 546 phalloidin (red actin myofibrils) and parasites expressing GFP are seen in the cytoplasm. This is a representative experiment of three independent experiments performed with similar results.

In order to investigate the role of the TcCRT expressed on the surface of the parasite in the process of host-cell infection, we exposed GFP-expressing trypomastigotes pre-incubated with anti-TcCRT IgG monovalent Fab fragments to either WT or TSP-1 KO MEF and evaluated the level of cellular infection. We observed that pre-incubation of GFP-expressing trypomastigotes with anti-TcCRT IgG Fab significantly decreases cellular infection of WT MEF as evidenced by the decrease in the relative fluorescence units (RFU) of transgenic parasites in WT MEF (200±18) RFU after 72 hours when compared to parasites pre-incubated with an isotype control Fab antibody (760±30) RFU (Figure 4A). This indicates that TcCRT expressed on the surface of the parasite plays an important role in cellular infection by *T. cruzi*.

In order to identify the region of the TSP-1 molecule that plays a role in the process of *T. cruzi* infection, we exposed WT or TSP-1 KO MEF to GFP-expressing trypomastigotes previously pre-incubated with recombinant human TSP-1, recombinant NTSP or the recombinant C-terminal domain of TSP-1, containing the last type II repeat domain, the three type III repeat domains and the carboxy-terminal domain designated E3T3C1 [26]. Pre-incubation of invasive trypomastigotes with TSP-1 significantly increased the infection of WT MEF (1080±20 RFU) compared to TSP-1 KO MEF (637±42 RFU) at 72 hours of infection (Figure 4B). Pre-incubation of parasites with NTSP caused a more pronounced enhancement effect of cellular infection in WT MEF (1306±40 RFU) compared to (750±53 RFU) in TSP-1 KO MEF (Figure 4B). In contrast, pre-incubation of parasites with the C-terminal domain of TSP-1, E3T3C1, did not significantly enhance cellular infection of WT MEF compared to TSP-1 or NTSP (Figure 4B). These results indicate that TSP-1 plays an important role in enhancing the cellular infectivity of *T. cruzi* and that the N-terminal domain of TSP-1 is critical in enhancing *T. cruzi* infection of WT MEF, compared to the E3T3C1. WT MEFs are significantly more infected by *T. cruzi* than TSP-1 KO MEF as evaluated by fluorimetric infection assays (Figure 4A, B) and visualized by fluorescence microscopy (Figure 4C). This indicates that the expression of TSP-1 in cells plays an important role in enhancing cellular infection by *T. cruzi*.

## Discussion

Previous studies in our laboratory showed that invasive *T. cruzi* trypomastigotes up-regulate the expression of TSP-1 in human coronary artery smooth muscle cells [3]. Furthermore, knockdown of TSP-1 rendered mammalian cells less susceptible to cellular infection by *T. cruzi* indicating that TSP-1 plays an important role in the process of cellular infection by *T. cruzi*. However, the mechanism by which TSP-1 is up-regulated by the parasite modulated cellular infection is not completely known. The elucidation of molecules on the surface of *T. cruzi* that enhance cellular infection will advance our understanding of the molecular pathogenesis of *T. cruzi* infection. We anticipated that the N-terminal domain of TSP-1, which is specific to this isoform of thrombospondin, would be essential in the interaction with the parasite because it is different for all thrombospondin isoforms compared to the conserved C-terminal domain [14]. In this study we show that TSP-1 interacts specifically with *T. cruzi* trypomastigote surface proteins to enhance the process of cellular infection. We used a protein-protein interaction pull down assay followed by mass spectrometric analysis to show for the first time that TSP-1 interacts with several proteins on the surface of invasive *T. cruzi* trypomastigotes including *T. cruzi* calreticulin, several *T. cruzi* heat shock proteins including *T. cruzi* HSP 60, 65 and other lower molecular weight proteins of about 30 and 36 kDa. In this study, we focused on TcCRT since it has been suggested that TSP-1 interacts with human calreticulin [26]–[29] to signal mammalian cells. Therefore our observations agree with previous findings that TSP-1 interacts with host CRT. The fact that several other proteins were pulled down in the protein-protein co-precipitation assays show that human TSP-1 may interact with more than one protein on the surface of invasive trypomastigotes including calreticulin or that TcCRT forms a complex with other parasite surface molecules that are pulled down. This finding is in agreement with others, which showed that human TSP-1 interacts with HuCRT and a co-receptor [27], [28]. NHS-biotin pre-labeling, which does not penetrate the parasite membrane, showed that *T. cruzi* has a 48 kDa TcCRT protein on its surface that interacts with TSP-1 and NTSP.

Our observation that TSP-1 interacts with TcCRT located on the membrane of the parasite is also in agreement with the contemporary knowledge that CRT, which was previously thought to be exclusively intracellular, is also expressed on the surface of the parasite [29]–[31]. The identification of CRT homologs in other parasites such as *Onchocerca*, *Schistosoma* and *Leishmania* [17], [32], [33] suggests that the protein functions as an intracellular chaperone and its role in the process of infection of those parasites remains unknown.

Here we show that surface TcCRT is a virulent factor that interacts with host TSP-1 to enhance cellular infection by *T. cruzi*. TSP-1 and NTSP interacted with a 48 kDa protein on the surface of *T. cruzi* trypomastigotes that was identified as TcCRT. This protein is of parasite origin because we showed in pulse chase experiments that a radiolabeled nascent parasite surface protein of 48 kDa interacted with both full length TSP-1 and NTSP. The interaction of the TcCRT with NTSP is supported by previous findings which showed that HuCRT interacts with the N-terminal domain of TSP-1 [29], [34]. The molecular interaction between TcCRT and TSP-1 remains unknown. The elucidation of the molecular interaction between the two molecules will enhance our understanding of the molecular pathogenesis of *T. cruzi* and also lead to the development of molecular strategies to reduce *T. cruzi* infection.

Anti-TcCRT monospecific polyclonal IgG antibodies interact strongly with TcCRT in total parasite lysate, solubilized parasite membrane proteins, TcCRT pulled down by TSP-1 or NTSP and with the surface of invasive *T. cruzi* trypomastigotes. This further confirms the presence of TcCRT on the surface of the parasite that interacts with TSP-1. Anti-TcCRT also shows a weak interaction with a protein of about 85 kDa which might be complexed TcCRT or a protein that shares a common epitope with TcCRT.

In order to explore the significance of host TSP-1 and TcCRT in cellular infection by *T. cruzi*, we used TSP-1 KO MEF and WT MEF in our infection assays. We showed that the presence of host TSP-1 and parasite surface TcCRT are important for MEF cellular infection by *T. cruzi*. The significance of surface TcCRT in enhancing cellular infection by *T. cruzi* was supported by the fact that specific antibodies to TcCRT significantly reduced cellular infection. The observation of *T. cruzi* infection in the absence of TSP-1 indicates that there are other mechanisms independent of TcCRT-TSP-1 that are involved in the process of *T. cruzi* infection; but this does not negate the importance of this interaction during *T. cruzi* infection. The identification of TcCRT as a virulent factor expressed on the surface of the parasite can be exploited to provide new insights into the molecular mechanisms of cellular infection by *T. cruzi*. TcCRT expression on the parasite surface may modulate the vertebrate complement system as an immune escape mechanism [22].

Exogenous TSP-1 significantly enhanced cellular infection of WT MEF compared to TSP-1 KO MEF. However NTSP, which also pulled down TcCRT, significantly enhances parasite cellular infection compared to full length TSP-1. This may be due to the less complex structure of NTSP in relationship to TSP-1. Exogenous TSP-1 does not completely restore the degree of cellular invasiveness of TSP-1 KO MEF to that of WT MEF possibly because the ECM that was formed from within the cell and exported in to the pericellular microenvironment was not properly modeled in the absence of TSP-1. The recombinant C-terminal domain of TSP-1 E3T3C1 [26], which does not specifically bind to the surface of the parasite, was not able to significantly enhance cellular invasion of MEF by *T. cruzi* as compared to NTSP and full length TSP-1 molecules. Since the N-terminal domain of TSP-1 is essential for enhancing the cellular infection of MEF by *T. cruzi* in the presence of TcCRT, this interaction may be further exploited to develop specific molecular intervention approaches to combat Chagas’ heart disease. Taken together, our findings indicate that TSP-1 interacts with TcCRT, a virulent factor, on the surface of *T. cruzi* through the NTSP domain and that this interaction enhances cellular infection by *T. cruzi*.

## Materials and Methods

### Ethics Statement

The animal study was carried out in accordance with the protocols approved by the Institutional Animal Care and Use Committee (IACUC) of Meharry Medical College. The approved protocol Numbers were 080125PN026 and 080125PN026-01.

The antibody production in goats was carried in accordance with protocols approved by GenScript (Piscataway, NJ).

### Tissue Culture

#### *T. cruzi* trypomastigotes

Pure cultured trypomastigotes of the highly infective trypomastigote clone MMC 20A of the Tulahuen strain of *T. cruzi* were harvested from the supernatant of heart myoblast monolayers as previously described [35], [36]. For the preparation of *T. cruzi* solubilized membrane proteins, the parasites were washed several times in PBS (Invitrogen, Carlsbad, CA) and 1×10^8^ parasites were resuspended in HEPES buffered saline, containing 2 mM CaCl_2_, 2% CHAPS and 10% protease inhibitor cocktail set III (Calbiochem, Gibbstown, NJ). The resuspended parasites were incubated with vigorous shaking at 4°C for 30 minutes, microfuged and the supernatant was collected. For the pulse chase experiment, *T. cruzi* trypomastigotes were washed with methionine free MEM and pulsed with ^35^S methionine for 2 hours at room temperature with gentle rocking. A portion of the labeled parasites was subjected to a second labeling with NHS-biotin (Invitrogen, Carlsbad, CA) as described by the manufacturer.

The parasites used in the infection assays were *T. cruzi* trypomastigotes clone MMC 20A expressing green fluorescent protein (GFP), which were produced as previously described [37]. The GFP-trypanosomes were washed in Dulbecco’s modified Eagle’s medium (DMEM) containing high glucose (Invitrogen, Carlsbad, CA) and resuspended at 1×10^7^ parasites/ml in DMEM supplemented with 1% crystallized probumin (Millipore, CA).

### Generation of Mouse Embryo Fibroblasts (MEF) and Infection Assays

The mice used for these studies were maintained and treated in accordance with a Meharry Medical College approved IACUC protocol. TSP-1 deficient (TSP1−/−) mice in the C57BL/6J background stock No. 006141 and wild type C57BL/6J mice (Jackson Laboratory, Bar Harbor, Maine) were used for the generation of MEF. MEF were generated as described [38].

For the infection assays, MEF were subcultured in 8-well labtech chamber slides (Fisher, Pittsburgh, PA), and exposed to GFP-expressing trypomastigotes that were pre-incubated with endotoxin free recombinant TSP-1, NTSP, E3T3C1, at a ratio of 10 parasites per cell in a total volume of 200 µl in complete high glucose DMEM media containing 10% FBS, 1% each of multivitamins, MEM amino acids and pen/strep (Invitrogen, Carlsbad CA) for 72 hours and quantified using a Synergy HT fluorometer (Biotek Instruments, Winooski, VT). For the antibody inhibition assays, the parasites were pre-incubated with 2.5 µg of IgG monovalent Fab fractions before being exposed to MEF monolayers. Unbound trypomastigotes were washed away with HBSS (Invitrogen, Carlsbad CA) and parasite infections of MEF were similarly quantified. For fluorescence microscopic observation of *T. cruzi* multiplication within MEF, the monolayers were fixed with 2.5% paraformaldehyde, perforated with PBS-0.1% Triton X-100 and stained with 4′, 6-diamidino-2-phenylindole (DAPI) at a dilution of 1∶4000 for 5 minutes at room temperature and washed to visualize the DNA. The cells were also stained with Alexa fluor 546 phalloidin (Invitrogen, Carlsbad, CA) as described by the manufacturer, to show actin myofibrils by microscopy.

### Binding of Labeled Proteins to *T. cruzi* Trypomastigotes

The recombinant proteins, anti-TcCRT IgG or control antibody isotype were labeled with Alexa fluor 488 using the protein labeling kit essentially as described by the manufacturer (Invitrogen, Carlsbad CA). *T. cruzi* trypomastigotes were plated onto CC2 labtech chamber slides (Fisher, Pittsburgh, PA), fixed with 2.5% paraformaldehyde, and blocked with 1% BSA in HBSS before exposure to the labeled proteins for 1 hour at room temperature. Unbound proteins were washed and the parasite DNA was stained with DAPI and washed before fluorescence microscopic visualization. For competitive binding assay, the parasites were preincubated with 100X unlabeled protein before exposure to the labeled protein. For fluorescence-activated cell sorter (FACS) analysis, pure cultured trypomastigotes (10^7^ parasites) were washed with PBS supplemented with 1% BSA, incubated with labeled TcCRT IgG or labeled isotype control in the wash buffer for 1 hour at 37°C. Then the parasites were washed again in PBS and fixed in 1 ml of PBS-4% paraformaldehyde. The parasite associated fluorescence was excited at 488 nm and quantified using a FACSCalibur (BD Biosciences, Sao Jose, CA, USA). Trypomastigotes incubated with control isotype antibody were also run in parallel. The FACS analysis was done at the Meharry FACS/BSL3 core facility.

### Parasite Protein-protein Interaction Assays and Mass Spectrometry

TSP-1, NTSP, BSA were coupled to M-280 Tosyl activated Dynabeads (Invitrogen, Carlsbad CA) as described by the manufacturer. The coupled beads were incubated with solubilized membrane proteins of invasive trypomastigotes on a revolving platform at 4°C overnight. The magnetic beads were washed several times with PBS and then finally with PBS containing 6 M urea pH 8.0. The parasite proteins interacting with the coupled proteins were boiled in Laemmli sample buffer before separation on a 12% polyacrylamide gel. Gel slices were cut for in gel digestion followed by LC-MS/MS at the proteomics core of Vanderbilt University, Nashville, TN (http://www.mc.vanderbilt.edu/root/vumc.php?site=msrc). Briefly, after staining with colloidal coomassie, TSP-1, NTSP interacting protein bands were excised and subjected to in-gel tryptic digestion. Resulting tryptic peptides were analyzed by LC-MS/MS analysis at the Vanderbilt University Mass Spectrometry Research Center using an LTQ-orbitrap mass spectrometer (ThermoFisher) coupled with a nano-electrospray ion source. A full scan mass spectrum followed by 5 data-dependent tandem mass spectra (MS/MS) were collected throughout the 90 minute run using dynamic exclusion to minimize acquisition of redundant spectra. MS/MS spectra were searched via SEQUEST against the TriTrpyDB *T. cruzi* protein database (Tcruzi_AnnotatedProteins-v2.3, http://TryTrypDB.org). A reversed decoy version of the *T. cruzi* protein database was created for each of the protein entries in order to determine peptide false discovery rates from the LC-MS/MS data. Results were filtered to a <5% peptide false discovery rate and collated at the protein level using IDPicker [39], [40] (http://www.ncbi.nlm.nih.gov/pubmed/19522537). These results were further filtered by only including proteins having 2 or more unique peptide spectral identifications, resulting in false positive rates of <1%.The bioinformatics analysis of the Mass spectrometry data were done at the Meharry Medical College Microarray & Bioinformatics Core facility. In order to ensure that the proteins binding to TSP-1 or NTSP were of parasite origin, we labeled the parasites with 1 mCi (37 MBq) ^35^S methionine (MP Biomedical, Solon, OH) for 2 hours at room temperature. The solubilized membrane fractions from labeled parasites were incubated with coated beads overnight, washed, eluted, transferred to nitrocellulose membranes and then exposed to X-ray film. For double labeling, some of the radioactively labeled parasites were further label with sulfo-NHS Biotin (Pierce, Rockford, IL) at 4°C as described by the manufacturer.

The solubilized double label membrane proteins of invasive trypomastigotes were incubated with the NTSP, TSP-1 or BSA coupled Dynabeads on a revolving platform at 4°C overnight. The magnetic beads were washed several times with PBS and then finally with PBS containing 6 M urea pH 8.0 before incubation with Dynabeads M-280 strepavidin for 1 hour at 4°C and wash with PBS. The double labeled parasite proteins interacting with the coupled proteins and streptavidin were boiled in Laemmli sample buffer before separation on a 12% polyacrylamide gel, transferred to NC membranes and developed by autoradiography.

### TcCRT Cloning, Purification of r-TcCRT and Trypanosome TcCRT Characterization

*T. cruzi* trypomastigote total RNA was purified using RNeasy Mini Kit (Qiagen, Valencia, CA). The total RNA was reverse transcribed to cDNA using SuperScript® III Reverse Transcriptase (Invitrogen, Carlsbad, CA) and TcCRT cDNA was PCR amplified. The forward primer *catatg*cgtgcagcaatttttttctgtgc and the reverse primer: *aagctt*ttacaaatcctccttatcacggtcac were used with an initial Tm of 57°C. The attached *Nde* I and *Hind* III sites added to the forward and reverse primers respectively, facilitated directional cloning of the PCR product into the pGS21a vector (GenScript, Piscataway, NJ) which has a 6X Histidine N-terminal tag for affinity protein purification. The recombinant plasmid was used to transform BL21 star competent cells and the encoded protein expressed in overnight expression broth as described by the manufacturer (Novagen, EMD Chemicals, Gibbstown, NJ). The expressed His-tagged proteins were purified over a Ni-NTA affinity matrix (Qiagen). The purified recombinant TcCRT was used for the generation of monospecific polyclonal antibody in goats (Genscript, Piscataway, NJ). Platelet purified and recombinant TSP-1 (Protein Biosciences, Meriden, CT), recombinant NTSP and E3T3C1 were provided by Dr. Jack Lawler’s laboratory.

*T. cruzi* trypomastigotes total lysate, membrane proteins, rTcCRT, TSP-1 and NTSP pulldown were separated on a 4–20% SDS polyacrylamide gradient gel, transferred onto nitrocellulose membranes and probed with goat anti-TcCRT IgG (1∶10,000) followed by anti-goat HRP (1∶10,000). The protein bands interacting with the antibody were revealed using a chemiluminescence kit (GE Healthcare, Piscataway, NJ) as described by the manufacturer. Control blots were probed with an isotype control antibody.

Statistical Analysis: Data were analyzed with the student’s T-test. Statistical differences (p<0.05) are indicated on each figure using asterisk (*) to denote significant difference from control or TSP-1 KO MEF cells.
